# *Dioscorea nipponica* Makino: a systematic review on its ethnobotany, phytochemical and pharmacological profiles

**DOI:** 10.1186/s13065-018-0423-4

**Published:** 2018-05-11

**Authors:** Si-hong Ou-yang, Tao Jiang, Lin Zhu, Tao Yi

**Affiliations:** 1 School of Chinese Medicine, Hong Kong Baptist University, Kowloon Tong, Hong Kong Special Administrative Region China; 2 College of Chemistry, Leshan Normal College, Leshan, 614004 China

**Keywords:** *Dioscorea nipponica* Makino, Steroid saponins, Geographical distribution, Active ingredient, Pharmacological studies

## Abstract

*Dioscorea nipponica* Makino is a perennial twining herbs belonging to the family Dioscoreaceae, which is mainly distributed in the northeastern, northern, eastern and central regions of China. Traditionally, the rhizome of this herb has been commonly used by Miao and Meng ethnic groups of China to treat rheumatoid arthritis, pain in the legs and lumbar area, Kashin Beck disease, bruises, sprains, chronic bronchitis, cough and asthma. Modern pharmacological studies have discovered that this herb possesses anti-tumor, anti-inflammatory, anti-diuretic, analgesic, anti-tussive, panting-calming and phlegm-dispelling activities, along with enhancing immune function and improving cardiovascular health. In recent years, both fat-soluble and water-soluble steroidal saponins were isolated from the rhizomes of *D. nipponica* using silica gel column chromatography, thin layer chromatography and high performance liquid chromatography methods. Saponin and sapogenins are mainly responsible for most of the pharmacological effects of this plant. Further, the chemical components of the aboveground parts contain more than 10 kinds of phenanthrene derivatives. The present review summarizes the knowledge concerning the geographical distribution, chemical composition, pharmacological effects, toxicology studies and clinical applications of *D. nipponica*. 
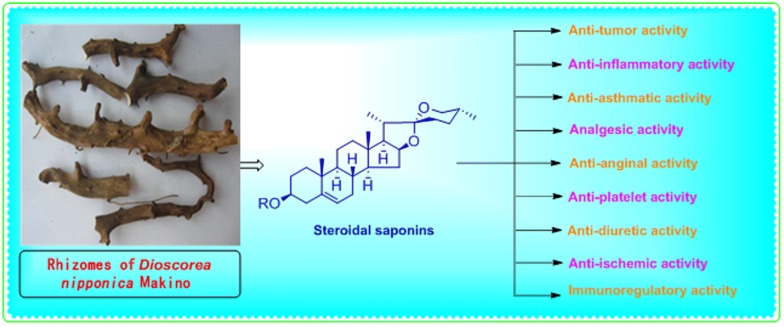

## Introduction

*Dioscorea nipponica* Makino is a species of the Dioscorea genus belonging to the family Dioscoreaceae. According to the traditional Chinese medicine, it is bitter-sweet in taste and warm in nature [1–7]. It mainly acts on liver, kidney and lungs, displaying anti-rheumatic, analgesic, blood circulation-stimulating, the lung channel-dredging, digestive, anti-diuretic, anti-tussive, panting-calming and phlegm-dispelling activities [2, 3, 5, 6]. Medicinally, it is commonly used for the treatment of rheumatoid arthritis, pain in the legs and lumbar area, Kashin-Beck disease, bruises, sprains, chronic bronchitis, cough and asthma [2, 5, 6]. In recent times, it has been used as an important industrial raw material for the synthesis of steroid hormones and saponin drugs for coronary heart disease [2].

## Geographic distribution

**Fig. 1:**
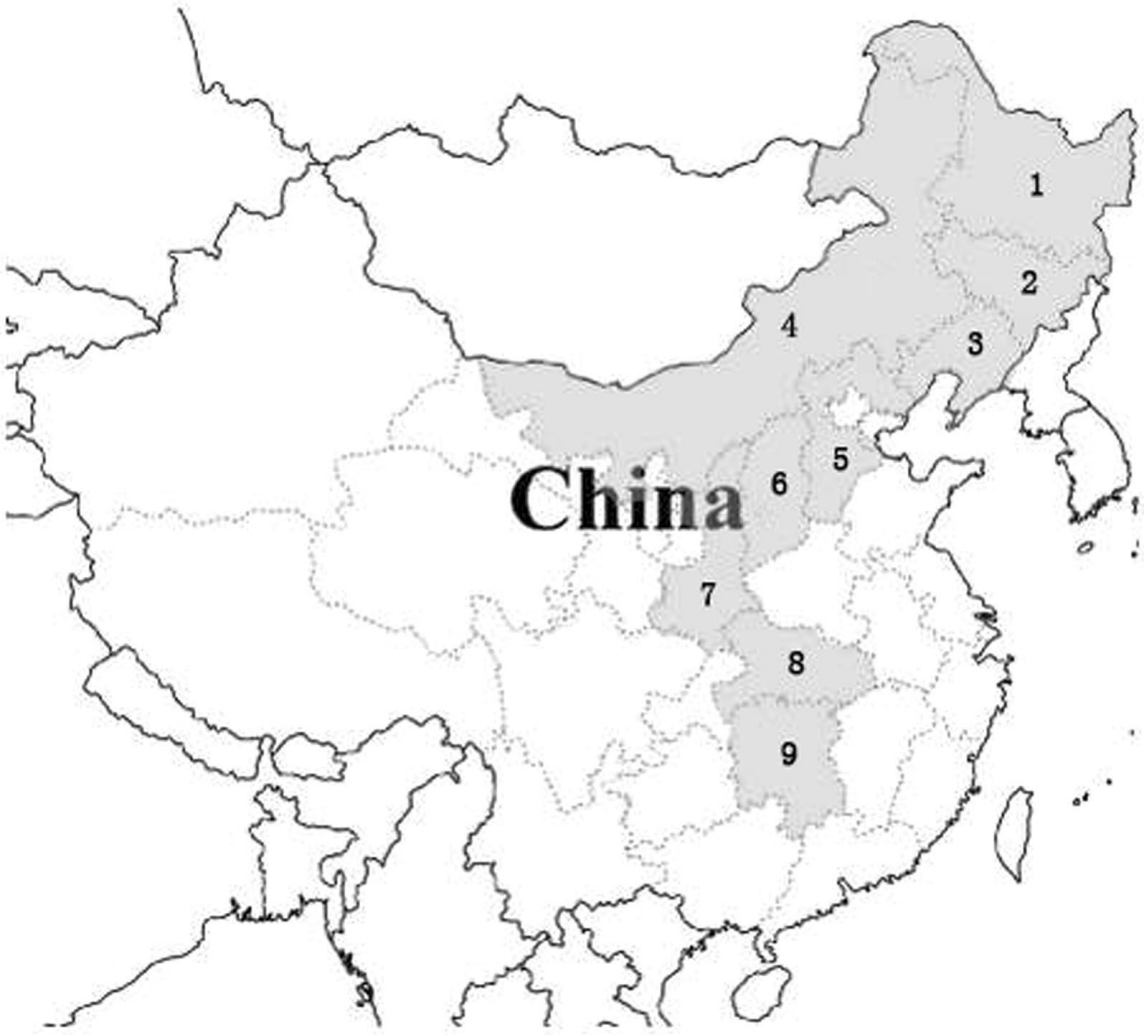
Distribution of *D. nipponica* in China: (1) Heilongjiang, (2) Jilin, (3) Liaoning, (4) Neimenggu, (5) Hebei, (6) Shanxi, (7) Shanxi (the north of Qinling), (8) Hubei, and (9) Hunan provinces

*Dioscorea nipponica* is a perennial twining herb, which grows in the north and central subtropical zones. It is distributed in the range of East longitude 105° ~ 109° to North latitude 26°34′ ~ 50°15′ [8], mainly in the northeastern, northern, eastern and the central regions of China, such as Shandong, Henan, Anhui, the north of Zhejiang, Jiangxi (Lushan), Shanxi (the north of Qinling), Gansu, Ningxia, the south of Qinghai, the northwest of Sichuan [2, 9, 10], Heilongjiang, Jilin, Liaoning, Shanxi, Henan, Shanxi, Hubei, Gansu, Hunan, Jiangsu and Zhejiang (Fig. 1) [3, 9, 11, 12]. It is grown in Tianshui, Longnan, Pingliang, Qingyang, Dingxi, Gannan, Linxia [13] and also in the north of Honshu as well as Korea and eastern Russia [2]. Changbai Mountain in the northeastern China is one of the major distribution and resource supply areas of *D. nipponica.* In addition, the wild resources are scattered vertically at the altitude range of 100–1000 m in Korea, Japan, Russia and other countries [8, 14, 15].

## Botanical description

**Fig. 2:**
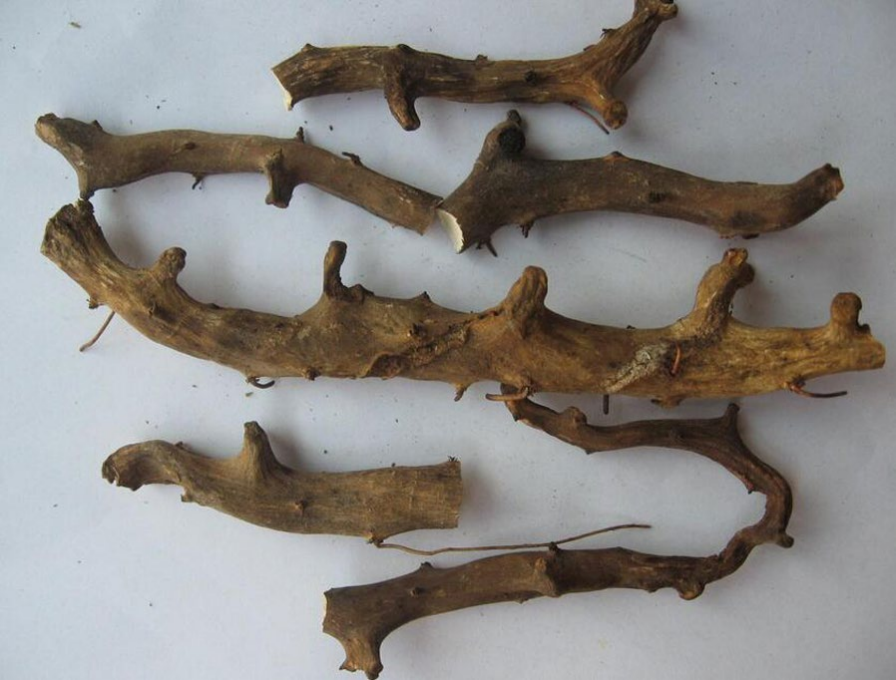
Photos of the rhizomes of *D. nipponica*

**Fig. 3:**
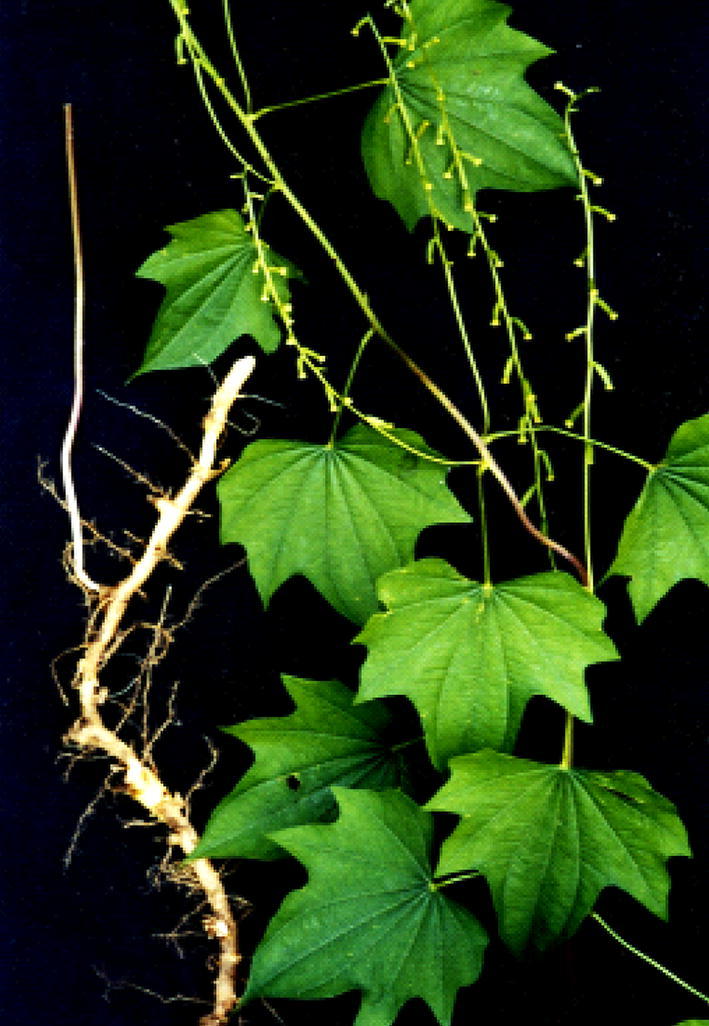
Photo of the plant of *D. nipponica*

*Dioscorea nipponica* is a perennial twiner, whose length can reach up to 5 m. The rhizome is horizontal, woody, highly branched, has a slightly curved cylindrical shape and its cross section appears white or yellowish-white with light brown vascular bundle dots [8]. In addition, its phellem layer is obviously detached and its tawny cortex falls off as flakes (Fig. 2) [16]. The aerial stem is slender, cylindrical, glabrescent with longitudinal grooves and twines to the left. The leaves are alternate simple, and palmate with a length of 10–15 cm and a width of 9–13 cm. The petiole length ranges from 10 to 20 cm while the leaf edge has an anisometric triangular shallow, medium or deep crack. The apex leaves are smaller, nearly entire, yellowish-green, shiny, glabrous or sparsely minutely setose, especially thick on the pulse (Fig. 3).

The flower of *D. nipponica* are unisexual, yellowish-green and droop like a small bell. The plant is dioecious; the male inflorescence is an axillary compound spike usually composed of 2–4 integrated small flowers and has a simple flower with lanceolate bracts at the top. The saucer-shaped perianth is 6-cracked with obtuse apex lobes, while 6 stamens are inserted at the middle with inward anthers. The female inflorescence is spicate, solitary; perianth is 6-cracked with lanceolate lobes; stigma is 3-cracked and the lobes are 2-cracked [17, 18].

The mature dry capsules are yellow, obovate-elliptic and prismatic in shape with winged edges; with a usual length of about 2 cm and a width of 1.5 cm. There are two rectangular seeds surrounded by rectangular wings in each locule inserted at the base of the axis, in which sometimes only one is fertile [19]. Moreover, the flowering period is from June to August and the fruiting stage is from August to October [20–24].

## Phytochemistry

Du et al. [25] employed separation methods such as silica gel column chromatography, thin layer chromatography (TLC) and high performance liquid chromatography (HPLC) to characterize the chemical constituents of *D. nipponica*. The main bioactive compounds of *D. nipponica* are steroid saponins which can produce aglycones and sugars after hydrolysis. The structures of the aglycones are composed of three types of scaffolds: furostane, a pentacyclic ring system with a sixth open ring; spirostane, a hexacyclic ring system; and pregnane, a tetracyclic ring system. The sugar part is mainly composed of glucose and rhamnose in various proportions and linkages. The saponins of this plant mainly include both water-soluble and water-insoluble steroid saponins. The water-soluble saponins mainly include dioscin, gracillin and *Trillium tschonoskii* saponin. On the other hand, the content of diosgenin, the major steroid sapogenin, in *D. nipponica* is 1.5–2.6% [21]. In the water-insoluble saponins, the sugar part comprises of 3-*O*-{*α*-l-rhamnopyranosyl-(1 → 2)-[*β*-d-glucopyranosyl-(1 → 3)]}-*β*-d-glucopyranoside; while the water-soluble ones possess 3-*O*-[*α*-l-rhamnopyranosyl-(1 → 3)-*α*-l-rhamnopyranosyl-(1 → 4)-*α*-l-rhamnopyranosyl-(1 → 4)]-*β*-d-glucopyranoside.

Li et al. [26] analyzed the saponin components by using the HPLC–ESI–MS/MS method and characterized five compounds including dioscoresides A, dioscoresides C and hypoglaucine A. By adopting the method of silica gel column chromatography, Kang et al. [27] separated new furan gonane saponins from the rhizome, namely, 26-*O*-*β*-d-glucopyranosyl-25(*R*)-22-hydroxy-furostanol-Δ5(6)-en-3*β*,26-dihydroxyl-3-*O*-{*α*-l-rhamnopyranosyl-(1 → 2)-*α*-l-rhamnopyranosyl-(1 → 4)}-*β*-d-glucopyranoside and 26-*O*-*β*-d-glucopyranosyl-25(*R*)-22-hydroxy-furostanol-Δ5(6)-en-3*β*,26-dihydroxyl-3-*O*-{*α*-l-rhamnopyranosyl-(1 → 2)-*α*-l-rhamnopyranosyl-(1 → 3)}-*β*-d-glucopyranoside.

Zhang et al. [28] carried out research on the water-soluble compounds of *D. nipponica* and identified 14 compounds, including 3 steroidal saponins, 3 phenolic glycosides, 2 dipeptides and 6 other classes of compounds. Shu et al. [29] separated 14 compounds, including 12 steroidal saponins and 2 diphenyl heptanes, among which, most of the steroidal saponins showed strong anti-tumor activity. Besides steroidal saponins, *D. nipponica* also contains water-soluble polysaccharides and other components. Wang et al. [30] extracted crude polysaccharides from this plant by applying the methods of hot water boiling and the ethyl alcohol precipitation. Li et al. [31] took the lead to separate an acidoid with strong anti-tussive effect from the water-soluble components, which is named as pair-acrinyl tartaric acid. Zhang et al. [32] identified at least eighteen amino acids in *D. nipponica*, and the total amino acid content accounts to 6.804%.

### Roots and rhizomes

**Fig. 4:**
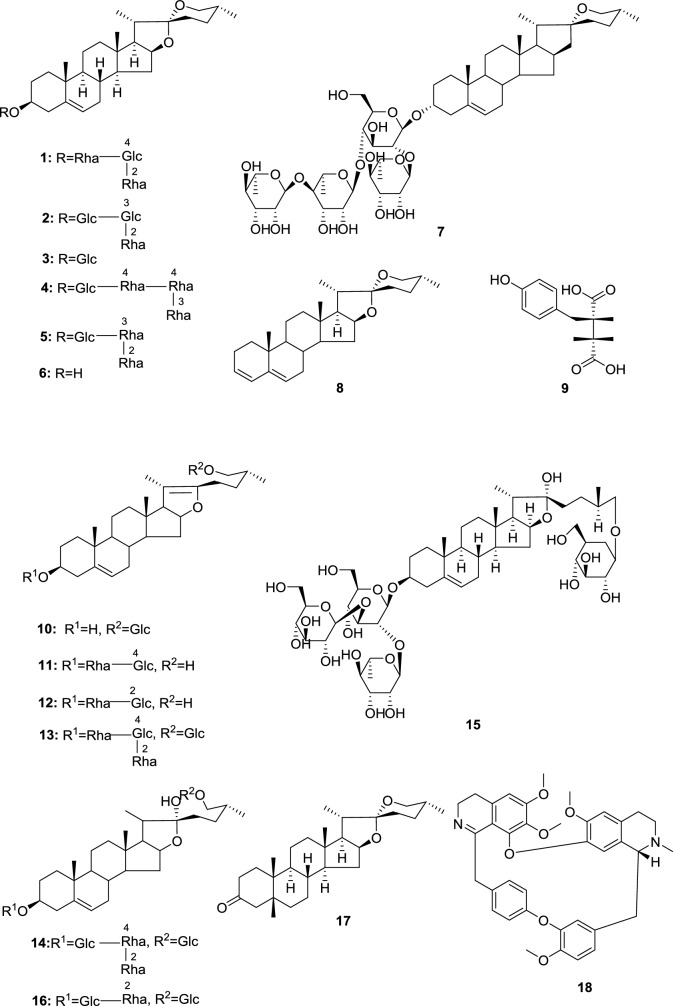
Chemical composition of *D. nipponica* roots (Glc = *β*-d-glucopyranosyl, Rha = *α*-l-rhamnopyranosyl)

Steroidal saponins are abundant in the roots and rhizomes of *D. nipponica*, where the following chemical compositions have been reported (Fig. 4): Dioscin (**1**) [9, 13, 33–47], gracillin (**2**) [9, 13, 33–36, 40, 42, 45–48], trillin (**3**) [9, 14, 36, 42, 44, 45, 49, 50], diosgenin-3-*O*-[*α*-l-rhamnopyranosyl-(1 → 3)-*α*-l-rhamnopyranosyl-(1 → 4)-*α*-l-rhamnopyranosyl-(1 → 4)]-*β*-d-glucopyranoside (**4**) [9, 25, 45], diosgenin-3-*O*-{*α*-l-rhamnopyranosyl-(1 → 2)-[*α*-l-rhamnopyranosyl-(1 → 3)]}-*β*-d-glucopyranoside (**5**) [25, 48, 51], diosgenin (**6**) [9, 36, 38, 42, 45–47, 49, 52], asperin (**7**) [9, 13, 45], 25-Δ-spirosta-3,5-diene (**8**) [9, 13, 14, 45–47, 53, 54], piscidic acid (**9**) [9, 13, 45–47, 55], 3*β*,26-glycol-25(*R*)-Δ5,20(22)-diene-furostanol-26-*O*-*β*-d-glucopyranoside (**10**) [40, 45, 56], progenin II (**11**) [9, 48, 52], prosapogenin A of dioscin (**12**, progenin III) [9, 48], pseudo-protodioscin (**13**) [9, 39, 40], protodioscin (**14**) [9, 36, 39, 40, 42, 48, 52], protogracillin (**15**) [9, 36, 40, 42, 45, 48], kikuba-saponin (**16**) [9], smilagenone (**17**) [45], and epistephanine (**18**) [45, 46]. In addition, allantoin, sterols, flavones, resins, polysaccharides and starch have also been identified as constituents in the roots of *D. nipponica* [14, 18].

### Aboveground parts

**Fig. 5:**
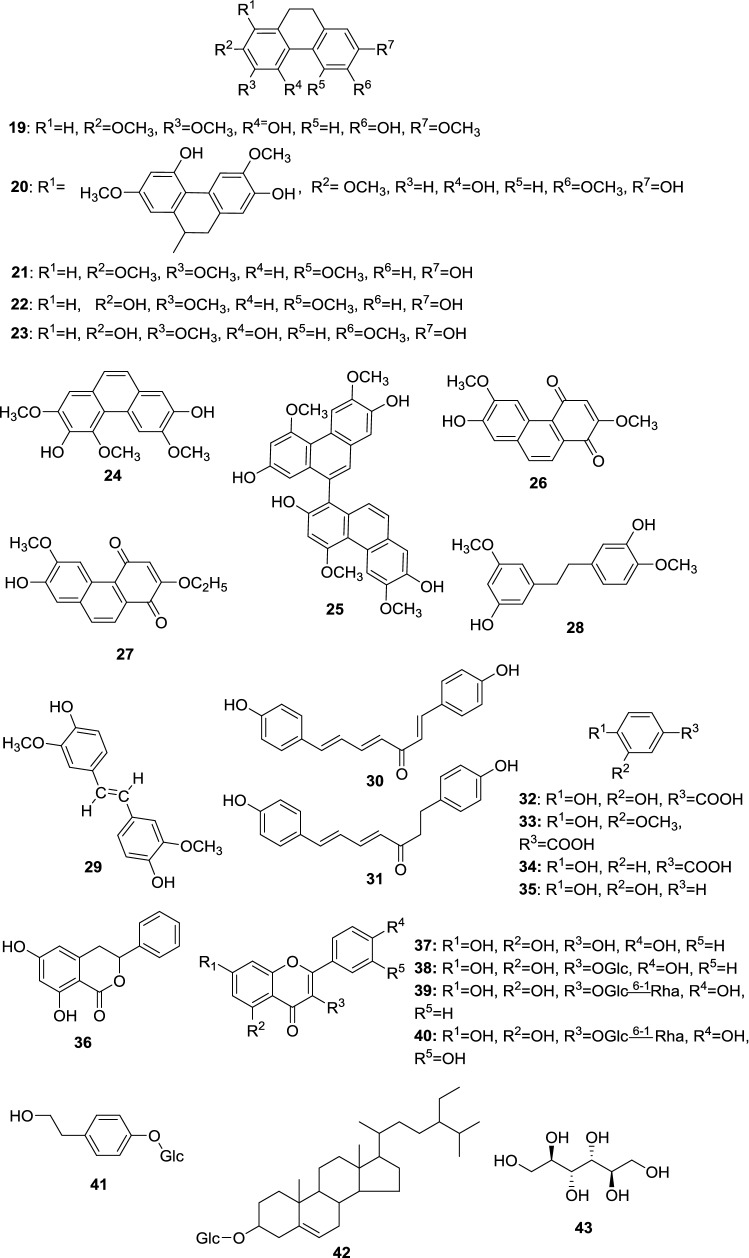
Chemical composition of the aboveground parts of *D. nipponica* (Glc = *β*-d-glucopyranosyl, and Rha = *α*-l-rhamnopyranosyl)

The classes of chemical constituents occurring in the aboveground portion of *D. nipponica* are presented in Table 1 and the structures are shown in Fig. 5.

**Table 1:** The chemical constituents occurring in the aboveground portion of *D. nipponica*

Class	Chemical compounds	Refs.
Phenanthrene-based compounds	4,6-Dihydroxy-2,3,7-trimethoxy-9,10-dihydrophenanthrene (**19**)	[57]
	1-(4,7-Dihydroxy-2,6-dimethoxy-9,10-dihydrophenanthrenyl)-4,7-dihydroxy-2,6-dimethoxy-9,10-dihydrophenanthrene (**20**)	[57, 58]
	7-Hydroxy-2,3,5-trimethoxy-9,10-dihydrophenanthrene (**21**)	[57]
	2,7-Dihydroxy-3,5-dimethoxy-9,10-dihydrophenanthrene (**22**, 6-methoxycoelonin)	[57, 59]
	4,7-Dihydroxy-2,3,6-trimethoxyphenanthrene (**23**)	[57, 60]
	3,7-Dihydroxy-2,4,6-trimethoxyphenanthrene (**24**)	[57]
	1-(2,7-Dihydroxy-4,6-dimethoxyphenanthrenyl)-2,7-dihydroxy-4,6-dimethoxyphenanthrene (**25**)	[57]
	7-Hydroxy-2,6-dimethoxy-1,4-phenanthraquinone (**26**, dioscoreanone) 2-ethoxy-7-hydroxy-6-methoxy-1,4-phenanthraquinone (**27**)	[57]
Phenols and organic acidic compounds	3′,5-Dihydroxy-3,4′-dimethoxybibenzyl (**28**)	[57]
	4,4′-Dihydroxy-3,3′-dimethoxy-trans-stilbene (**29**)	[57, 61]
	1,7-bis(4-Hydroxyphenyl)-1,4,6-heptatrien-3-one (**30**)	[57, 59, 61]
	1,7-bis(4-Hydroxyphenyl)-4,6-heptabien-3-one (**31**)	[57, 61]
	3,4-Dihydroxybenzoic acid (**32**, protocatechuic acid)	[57, 61]
	4-Hydroxy-3-methoxybenzoic acid (**33**, vanillic acid)	[57, 61]
	4-Hydroxybenzoic acid (**34**)	[57, 61]
	Pyrocatechol (**35**)	[57, 61]
Coumarins	(3*S*)-6,8-dihydroxy-3-phenyl-3,4-dihydroisocoumarin (**36**, montroumarin)	[57, 58]
Flavonoids	Kaempferol (**37**)	[57, 61]
	Kaempferol-3-*O*-*β*-d-glucopyranoside (**38**)	[57, 58]
	Kaempferol-3-*O*-*β*-rutinoside (**39**)	[57, 58]
	Quercetin-3-*O*-rutinoside (**40**, rutin)	[57, 61]
Glycosides	4-Hydroxyphenethyl-ol-4-*O*-*β*-d-glucopyranoside (**41**, icariside D2)	[57, 61]
Steroids	Daucosterol (**42**)	[57, 58]
Polyols	d-Mannitol (**43**)	[57, 58]

## Pharmacological activity

The rhizomes of *D. nipponica* (Rhizoma *Dioscorea nipponica* (RDN)) are rich in the dioscin content. After the oral administration of dioscin, diosgenin produced from the metabolism of intestinal flora is the major true bioactive compound. As verified by modern research, RDN has various pharmacological activities such as improving the cardiovascular system, regulating immune function, preventing tumors, phlegm, cough and asthma, as well as relieving inflammation and pain [62].

### Effects on the cardiovascular system

Dioscin can improve myocardial injuries, prevent the anti-oxygenation of myocardial cells, relieve the calcium overload of myocardial cells, and prevent the hypoxia of myocardium via different mechanisms [63].

Through the hypoxia/reoxygenation damage model of the neonatal rat in vitro cardiomyocyte culture, Ni et al. [64] compared the levels of superoxide dismutase (SOD), malondialdehyde (MDA) and nitric oxide (NO) in the control group, the hypoxia/reoxygenation injury group, and the dioscin groups (10, 30, and 100 mL/L, respectively). In this context, dioscin significantly improved the in vitro SOD activity of myocardial cells after hypoxia/reoxygenation and reduced the content of MDA and NO in a dose-dependent manner. This indicated that dioscin can play a protecting role in the hypoxia/reoxygenation of myocardial cells through the anti-oxygenation effect. Using a similar model, Gao et al. [65] concluded that the myocardial cell beat rate, cell survival rate, and mitochondrial membrane potential of the dioscin intervention group were significantly higher than those in the ischemia–reperfusion (I/R) group. Moreover, the average calcium-ion fluorescence intensity in cardiac cells was clearly lower than that of the I/R group.

Fan et al. [66] observed a significant decrease in the ATP and adenylic acid levels in the myocardium of I/R rats using the Reversed-phase HPLC (RP-HPLC) method. In this context, the I/R rats with dioscin intervention have shown an increase in the ATP and adenylic acid levels, which further improved the myocardial energy metabolism disorder.

The expression percentages of CD62P and CD63 in plasma were detected using flow cytometry (FCM) while the platelet-activating factor (PAF) levels were determined through enzyme-linked immunosorbent assay (ELISA) method by Wei et al. [67]. The myocardial I/R model of dioscin-pretreated rats were studied, which revealed a significant reduction in the cytokine content of platelet activation in plasma. Hence, it was inferred that the mechanism of anti-myocardial I/R might be related to the anti-platelet activation.

Ning et al. [68] have observed that diosgenin could significantly reduce the pathological expression and degree of myocardial ischemia, reduce myocardial infarction, dilate the coronary arteries, increase myocardial blood supply, and improve the function of vascular endothelial cells in the acute dog experimental myocardial ischemia model. Based on this work, the influence of diosgenin on the cardiac hemodynamics of dogs was studied [69] and the results indicated that the former can dilate the coronary vessels as well as effectively reduce the coronary resistance and heart load. Meanwhile, there was an increase in cardiac output, cardiac stroke volume, myocardial blood and oxygen supply, while the effective impact on the heart can be strengthened. In this way, it can play the role in regulating and improving the cardiovascular system and providing an experimental basis for the clinical treatment of ischemic heart disease. Subsequently, the same group [70] used the coronary artery ligation myocardial infarction model of rats to measure the myocardial infarction scope and vasoactive effect of diosgenin. The result demonstrated that diosgenin could significantly reduce the extent of myocardial infarction, inhibit the increase of serum creatine kinase (CK) and lactate dehydrogenase (LDH), decrease the elevated MDA levels, increase the SOD and NO levels, reduce the level of myocardial enzymes, and enhance the oxygen free radical scavenging capacity.

Zhang et al. [71] used the ^86^Rb trace method to observe the impact of RDN saponins on the myocardial blood flow of rats. The results showed that the ^86^Rb myocardial intake ability of rats has been increased by 9.46% for the 225 mg/kg group (P < 0.05), 18.46% for the 337.5 mg/kg group (P < 0.05), and 31.47% for the 675 mg/kg group (P < 0.01). This indicated that the perhexiline of RDN and the water-soluble saponins can significantly increase the myocardial blood flow, relieve myocardial anoxia, and promote the treatment of coronary heart disease as well as angina.

### Regulating the immune function

Gao et al. [72] used sheep red blood cell (SRBC) hemolysin antibodies to generate the dinitrofluorobenzene (2,4-DNFB)-induced delayed-type hypersensitivity test model in mice in order to observe the impact of *D. nipponica* on the cellular immune function. The result showed that intragastric administration (1 g/kg) of total saponins of *Rhizoma Dioscoreae nipponica* (TSRDN) could significantly reduce the generation of SRBC hemolysin antibody in mice compared with the prednisone (2 mg/kg) group (P < 0.05). Further, TSRDN inhibited the generation of SRBC hemolysin antibodies of mice and the delayed-type hypersensitivity caused by DNFB, which suggests its significant role in inhibiting the humoral immunity and cellular immune function.

Nan et al. [73] used the decoction of RDN to feed the mice for 7 days, which caused the atrophy of thymus (P < 0.01). Moreover, the positive rate of nonspecific acid esterase (ANAE) was reduced (P < 0.05), the delayed type hypersensitivity of skin was inhibited (P < 0.01), the formation of serum hemolysin was decreased (P < 0.01), the chicken erythrocyte cytophagy percentage by the mice enterocoelia cell and the phagocytic index were increased, and the enzyme content of serum bacteriolysis has increased as well. On the contrary, the impact on the spleen weight and structure was insignificant (P < 0.05). Thus, they concluded that RDN could inhibit both cellular and humoral immunity, but enhance the phagocytosis of macrophages.

Song et al. [74] used the alcohol extract of RDN on mice: At a low dose of RDN (1.25 mg/mL), the concanavalin A (ConA)-induced splenocyte proliferation of mice could be inhibited, while at a higher dose (5 mg/mL) it could be clearly inhibited rapidly (30 h). This implied that the inhibitory effect of RDN on cell proliferation was mainly in the excitation stage. The same research group [82] adopted the serum pharmacology method to study the impact of TSRDN on the ConA-induced splenic lymphocyte proliferation and the secretion of IL-2. The results of this study suggested that the drug serum of TSRDN can inhibit the IL-2 production and T-lymphocyte proliferation as well as the cellular immunity in mice.

Yu et al. [75, 76] studied the influence of TSRDN on the lipopolysaccharide (LPS)-induced splenic lymphocyte proliferation and the secretion of IL-6 by using serum pharmacology in mice. A 20% concentration of TSRDN could prevent the LPS-induced splenic lymphocyte proliferation and IL-6 generation, thus inhibiting the humoral immunity of mice.

Shu et al. [29] used the decoction of RDN to feed mice for 7 days, which caused the atrophy of thymus (P < 0.01), the reduction of ANAE positive rate (P < 0.05) and the inhibition of delayed-type hypersensitivity of skin (P < 0.01). The formation of serum hemolysin was reduced (P ≪ 0.01), but the impact on the spleen weight and structure was unclear (P > 0.05). Further, the chicken erythrocyte cytophagy percentage in the mouse peritoneal macrophages and the phagocytic index as well as the serum lysozyme content were increased (P < 0.01 for all). This indicated that the cellular immunity and humoral immunity could be inhibited by RDN; whereas the phagocytosis of macrophages could be enhanced.

### Anti-tumor activity

A number of reports have established the anti-tumor effects of RDN, dioscin and diosgenin on various tumor cells, and the anti-tumor mechanisms were discussed respectively [77].

Wang et al. [78] used four tumor cell strains (MCF, L929, A375-S2, HeLa) for the in vitro test and four mice portability tumors (S-180, HepA, U14, EAC) for the in vivo test of the anti-tumor effect of different concentrations of diosgenin. It was observed that diosgenin has a clear inhibitory effect on mice transplant tumors like S-180, HepA and U14 as well as the tumor cell strains like L929, HeLa and MCF.

Wang et al. [79] used the prosapogenin B (P.B) of dioscin on the human leukemia cell line K562 (10 μmol/L) for 24 h. The K562 cells displayed typical intranuclear chromatin condensation, nuclear segmentation, withering of cell volume, surface film bubbling of apoptotic cells, etc. The result showed that P.B could inhibit the proliferation of K562 cells by inducing apoptosis.

Hong et al. [80] tested the effect of diosgenin on the human osteosarcoma cell line (U-20S) and observed a strong inhibitory effect. In a subsequent research, they [81] used the tetrazole reduction method to observe the impact of diosgenin on the in vitro growth of tumor cells (U-20S, SGC-7901 and ACCM), as well as the normal cells (HUCB and hRPE). The results showed that diosgenin could inhibit the growth of both normal and tumor cells to different degrees; the dose for inhibiting normal cells was 2–11 times more than that for inhibiting tumor cells.

Several years later, the same research group [82] explored the mechanism of action of diosgenin on the in vitro growth of human osteosarcoma epithelial cells line (U-20S) via the tetrazole reduction method. The flow cytometry analysis revealed that diosgenin inhibited the U-20S cell cycle in the G_1_ phase. The result showed that diosgenin had a clear inhibitory effect on the growth of U-20S cells, which has a certain value for the cure of osteosarcoma.

Hou et al. [83] studied the impact of dioscin on the cell cycle of breast cancer cells: dioscin downregulated the values in the phase G_0_/G_1_ and phase-S and upregulated the values of apoptotic cells in the phase G_2_/M. This result indicated that breast cancer cell proliferation and their inhibition were related to the impact on the cell cycle and apoptosis induction. Later, Chen et al. [84] further explored the anti-tumor activity of dioscin through the portable mouse breast cancer animal model. It was observed that both the dosage and administration routes could affect the portable mouse breast cancer, and the effect of intraperitoneal injection was optimal. In later studies [85], the anti-tumor activity of dioscin on various solid tumor cells was studied by the in vitro cell culture technique, which provided the basis for further exploration of portable animal model studies, anti-tumor mechanism elucidation as well as new drug research and development.

Li et al. [86] studied the synergistic and toxicity-reducing effect of diosgenin on the chemotherapy drug, 1-(2-tetrahydrofuryl)-5-fluorouracil (FT-207). The results showed that the combination of diosgenin with FT-207 could significantly enhance the anti-tumor activity on the mouse gastric adenocarcinoma cell line (MFC) and improve the immune function of macrophages while the toxic and side effects could be reduced as well.

Chen et al. [87] observed the impact of sapogenins on the tumor weight gain of poorly differentiated human gastric gland cancer cell strain (MGC-803) in nude mice. The result showed that diosgenin [400, 200 and 100 mg/kg (ig)] had an obvious dose-dependent inhibitory effect on the tumor growth with an average anti-tumor rate of 44.9–64.5%.

In the papers of Li et al. [88] and Song et al. [89], the inhibitory effect of diosgenin on the cell division and colony formation of MGC-803 cell line was reported. The possible working mechanism was explored as well, i.e., diosgenin could directly inhibit the DNA synthesis of MGC-803 cells at low concentrations (3.750 and 7.500 mg/L) with a median inhibitory concentration (IC_50_) of 13.17 mg/L.

### Effects on the respiratory tract

Bronchial asthma is a chronic airway inflammation disease with the participation of various cells (eosinophils, mastocytes, T-lymphocytes, neutrophils, and airway epithelial cells) and cellular components. Its pathogenesis is complicated, including allergic inflammation, airway remodeling, airway hyperresponsiveness, etc.

Cai et al. [90] studied the effect of TSRDN on the Ovalbumin (OVA)-induced airway remodeling and α-smooth muscle actin (α-SMA) of specific-pathogen-free (SPF) level 50 BALB/c asthmatic mice. The mice were randomly divided into the blank group, model group, high-dose group, middle-dose group and the low-dose group of TSRDN. In the experiment (18–55 days), mice of the blank group and the model group were administered with normal saline gavage, while the drug groups were given 80, 40, and 20 mg/kg of TSRDN. At the end of the experiment, the lung tissue pathomorphism change was observed and the immunohistochemical method was adopted to test the α-SMA level. The results showed that TSRDN could inhibit the lung tissue expression of α-SMA in asthmatic mice, affect the skeletal muscle thickening, and improve the airway remodeling status.

Wang et al. [91] used the OVA-sensitization method to establish an asthma rat model along with grouping and drug administration for 7 days. The eosinophilic granulocyte (EOS) infiltration in bronchoalveolar lavage fluid (BALF) of rats and the lung tissue pathological changes like inflammation were observed. The results indicated that the EOS infiltration in the BALF and lung tissue, pathological changes of the lung tissue and the airway inflammation in the *D. nipponica* treatment groups was significantly lower than that in the model group.

Zhang et al. [92] adopted the traditional Chinese prescription for *D. nipponica* together with ligustrazine acupoint injection to manage and treat bronchial asthma. The results showed that the total effective rate of the treatment group was 92.42% and that the markedly effective rate was 56.06%, which were superior to 83.33 and 26.67% of the control group (P < 0.05). At the same time, the improvement of the pulmonary function, IgE and other detection indicators were clearly superior to those of the control group (P < 0.01). It was proved that the drug can inhibit the platelet aggregation and release, and has the effects of reducing phlegm, clearing away lung-heat and relieving asthma.

Into the ammonia-induced mouse coughing method, oral administration of total saponin, water-soluble or water-insoluble saponin, no. 1 molecular sieve and intraperitoneal injection of decoction can achieve a significant anti-tussive effect, but diosgenin and high-dose steroidal saponin were ineffective. Li et al. [31] have separated an acidoid from the water-soluble components, which has a strong anti-tussive effect. According to the mouse phenol red method, oral administration of total saponins, water-insoluble saponin, no. 1 molecular sieve or intraperitoneal injection of decoction can have a significant expectorant effect [31].

### Anti-inflammatory and analgesic activity

Rheumatoid arthritis (RA) is a chronic auto-immune disease manifested as joint synovitis, synovial membrane pannus formation, and cartilage as well as bone tissue erosion. Angiogenesis and pannus formation play the central role in promoting RA, which is in turn affected by vascular endothelial growth factors (VEGF). Several studies indicated that TSRDN could inhibit the abnormal expression of synoviocyte VEGF in collagen-induced arthritis (CIA) mice and relieve the angiogenesis and inflammatory response [93].

Lv et al. [94] studied the effect of *D. nipponica* treatment on the urate crystal-induced acute gouty arthritis in rats. 56 rats were randomly divided into the control group, model group, colchicine group, *D. nipponica* group A (15 g/kg/day), *D. nipponica* group B (12 g/kg/days), *D. nipponica* group C (6 g/kg/days) and *D. nipponica* group D (3 g/kg/day). Compared with the model group, the joint swelling in *D. nipponica* treatment groups at each time point had decreased to different extents. Therefore, moderate and high doses of *D. nipponica* have certain treatment effect on acute gouty arthritis, which may be realized through the inhibition of white blood cell generation and the secretion of the important inflammation transmitter IL-1β.

Dong et al. [95] discussed the effect of TSRDN on the synovium angiogenesis and VEGF expression of CIA rats. The study found that the levels of rat synovium microvessel density (MVD) and VEGF of the model group were significantly lower than that in the treatment groups, suggesting that TSRDN can relieve the angiogenesis and inflammatory reaction via the inhibition of abnormal VEGF expression. A few years later, the same group [96] established a CIA rat model and observed the influence of TSRDN on inflammatory cytokines in the serum of type-II CIA rats, and the possible working mechanism of TSRDN in curing RA was discussed. The result showed that TSRDN could significantly decrease the levels of TNF-α, IL-1β, and IL-6 in the serum of CIA rats, alleviate the pathological damage of synovial tissue and may play a role in the treatment of RA.

Tang [97] used xylene and carrageenan to induce inflammation and conducted the mouse writhing reaction test. It was observed that *D. nipponica* could significantly inhibit the xylene-induced ear inflammation and carrageenan-induced ankle swelling in rats, reduce the permeability of mouse abdominal cavity capillaries and inhibit the cotton ball granuloma in rats. Furthermore, it can prolong the pain reaction time and reduce the writhing time in mice. Thus, it was concluded that *D. nipponica* had obvious anti-inflammatory and analgesic effects.

Yao et al. [98] studied the effect of *D. nipponica* on microcrystalline sodium urate-induced acute gouty arthritis of rats and oteracil potassium-induced hyperuricemia model of mice. The screening of pharmacodynamic indices, such as anti-inflammatory, analgesic, uric acid decrease, etc. was performed. A 30% alcohol elution of the *D. nipponica* water extract significantly reduced the blood uric acid level of mice with hyperuricemia, improved the pathomorphological change of the joint synovium tissue of rats, and decreased the writhing times; so, it was considered as an effective treatment for acute gouty arthritis.

Tong et al. [99] adopted the mouse peritoneal capillary exudation and cotton ball granuloma methods to compare the anti-inflammatory effects of the aboveground and the rhizome water extracts of *D. nipponica* (WERDN). The results showed that WERDN significantly reduced the exudation of peritoneal fluid and the weight of granuloma in mice, thus inhibiting the increase of early blood capillary exudation and the proliferation of late granulation tissue. The same research group [100] carried out a comparative research on the anti-inflammatory effect of aboveground and WERDN collected at the same time and in the same place by using the auricle swelling experiment, cotton ball granuloma and peritoneal fluid methods in mice. For the mouse cotton ball granuloma model, the intragastric administration of TSRDN for 7 days significantly alleviated the ear swelling, reduced the weight of granuloma, and decreased the peritoneal fluid in exudation. These results suggested that TSRDN had a clear anti-inflammatory activity.

Pang et al. [101] established a carrageen-induced acute non-infection inflammatory model of podedema of mice. 3 days before modeling, different doses of *D. nipponica* decoctum were used for gavage once per day. In the simulation, serum sialic acid levels and the thickness of foot pad was measured every 2 h before and after the modeling. It was concluded that *D. nipponica* could significantly alleviate the carrageen-induced swelling of the foot in mice, and it has no impact on the serum sialic acid levels. Du et al. [102] used the rat CIA model to study the therapeutic effect *D. nipponica*. Intragastric administration of *D. nipponica* water decoction reduced the CIA ankle swelling rate and improved the ankle pathological state.

Xie et al. [103] adopted TSRDN gavage treatments in various doses to improve the joint inflammatory symptoms of rats with adjuvant arthritis. The treatment caused a reduction in joint swelling, inflammation index, inflammatory reaction morbidity, cartilage damage and the degree of severity.

### Other effects

The pharmacological studies have confirmed that the *D. nipponica* extract or its components have effects of anti-aging [104], blood fat-reducing [105, 106], thyroid-regulating and anti-platelet aggregation.

#### The effect on the thyroid

Wang et al. [107] used the *D. nipponica* extract on Graves’ disease (toxic diffuse goiter) rat model and concluded its anti-thyroid activity. On the basis of this work, sodium-iodide symporter (NIS) mRNA expression was measured by similar means, and the result showed that *D. nipponica* could strongly inhibit the expression of NIS mRNA, thus inhibiting the effect of iodine capture [108]. After a 1-month gavage of *D. nipponica*, the electron microscopy technique was used to observe ultrapathological changes of rats’ thyroid tissue which revealed an improvement in the hyper-pathological disorder of the thyroid tissue in Graves’ disease [109].

#### The effect on platelet aggregation

Ning et al. [110] observed that diosgenin could inhibit the thrombosis of rats in vitro and vivo, could reduce the dry and wet weight of thrombus, prolong the thrombosis time in vivo, and reduce the whole blood viscosity as well as plasma viscosity. As a result, it provided an experimental basis for the clinical treatment of ischemic heart diseases and cerebrovascular diseases.

## Clinical applications

The dry rhizome of *D. nipponica* has the effect of expelling wind-damp; relieving pains; relaxing muscles and stimulating blood circulation; helping digestion and promoting urination; relieving cough, asthma and eliminating phlegm. It can be used for the treatment of rheumatoid arthritis, lumbar and leg pains, traumatic injuries, chronic bronchitis, and tumors. At present, the clinical application of the rhizome of *D. nipponica* reported in the literature is mainly concentrated on the following aspects:

### The treatment of arthritis

#### The treatment of rheumatoid arthritis

45 cases (35 male and 10 female) with rheumatoid arthritis were treated with intramuscular injection of *D. nipponica* (1 g/mL) twice a day. 84% of the patients’ treatment process took a month and 16% of the patients’ treatment took 2 months. The obvious effective, improvement and the total effective rates were 40, 43 and 83%, respectively. There were no significant adverse reactions, and the short-term effect was positive. 222 cases with rheumatic and rheumatoid arthritis were treated by administering *D. nipponica* (150 g) and dog bone (100 g) with the obvious effective, effective and total effective rates of 65.87, 27, and 92.7%, respectively [57, 111]. The prescription was composed of the *D. nipponica*, *Caulis spatholobi* and *Zingiberis* rhizome. It was used to treat rheumatic arthralgia and had the effects of dispelling wind and eliminating dampness, promoting blood circulation to remove the meridian obstruction, and eliminating cold to stop pain [112].

*Dioscorea nipponica* (10–15 mL) extract was administered orally thrice a day for 15 days and the symptoms could be relieved after only 7 days. 24 cases with brucellosis combined rheumatic arthritis were treated by using the *D. nipponica* injection (anti-“O” 1: over 400). The anti-“O” of 15 cases has decreased after treatment, of which 11 cases were negative. Thus, it was considered to be related to *D. nipponica* effect of inhibiting the streptococcus as well as the steroid hormones [113].

#### The treatment of osteoarthritis

Osteoarthritis is a common disease among the middle-aged and old populace, leading to chronic cartilaginous damage. The intraosseous hemodynamic abnormality that is characterized by venous blood stasis and corresponding high intraosseous pressure may be one of the main causes for the occurrence of the disease. In a study, *D. nipponica* decoction was used to treat 35 cases with knee osteoarthritis at 1 dose/day during a course of 30 days, where the degree of arthralgia, joint pressing pain, the degree of joint swelling and joint function grading were regarded as the observation indices. The total effective rate of the treatment course was 94.3% (P < 0.05) which indicated that *D. nipponica* had a good curative effect on osteoarthritis of the knee joint [114].

Among 128 cases of patients with knee osteoarthritis orally taking traditional Chinese medicine along with the external application of the bone spur medicine containing *D. nipponica*, 61 cases had been cured (47.7%), 38 cases had an obvious positive effect (29.7%), 23 cases had a positive effect (18%), and the total effective rate was 95.4% [115].

#### The treatment of acute suppurative arthritis

Among eight cases of acute suppurative arthritis resistant to antibiotics, six were with pyemia (*Staphylococcus aureus* in blood culture). After treatment with *D. nipponica* decoction (adults: 150 g; children: 100 g) twice a day, five cases have been cured [116].

### The treatment of coronary heart disease and angina

Coronary heart disease is one of the most common diseases that threaten health and life. Its major risk factors include high blood pressure, dyslipidemia, overweight/obesity, hyperglycemia/diabetes, unhealthy lifestyles such as smoking, irrational diet (high fat, high cholesterol, high calorific value, etc.), lack of physical activity, excessive drinking, and social psychological factors. Therefore, lipid-lowering, anti-coagulation, blood viscosity improvement and coronary blood flow volume increase are the key criteria for the treatment.

Diao xin xue kang capsules made from the rhizome extracts of *D. panthaica* or *D. nipponica* can promote blood circulation and remove blood stasis, dilate coronary blood vessels, improve myocardial ischemia, etc. It can be used to prevent and cure coronary heart disease, angina, chest apoplexy, dizziness, and shortness of breath, palpitations, chest distress or pain caused by internal/external bleeding [117].

The Weiaoxin group (treatment) contains 33 cases (20 male and 13 female), and Diao xin xue kang group (control) has 32 cases (24 male and 8 female). For 4 weeks, the treatment group took Weiaoxin pills (160 mg) and the control group took Diao xinxuekang capsules (200 mg) orally, thrice a day. During the period of observation, low fat and low salt diet were applied for the cases with high blood pressure and other relevant drugs except the quick-acting nitroglycerin were stopped. Both Weiaoxin and Diao xin xue kang had a similar effect on alleviating angina and reducing nitroglycerin dosage, but the total effect on improving electrocardiography, coronary and myocardial blood flow by Weiaoxin was superior to that of Diao xinxuekang (P < 0.05) [118].

Zhang et al. [119] used Weiaoxin pills to study the prevention and treatment of vasculopathy in diabetic patients. Weiaoxin pills (160 mg, 3 times a day) were used for 30 days in a row and then the dose was reduced (80 mg, 3 times/day). After administering for another 30 days, the thromboxane B2 (TXB2) in the serum, 6-ketone-prostaglandin F1α (6-Keto-PGF1α) content and hemorheology were tested. The results showed that Weiaoxin pills could reduce the whole blood viscosity, plasma viscosity and fibrinogen to different extents, decrease the blood concentration coefficient and the microcirculation detention times. So, its effect was significantly better than the conventional treatment group, indicating that oral administration of Weiaoxin pills in the early stages can effectively prevent diabetes and diseases of blood vessels.

Chuanlong guanxinning tablets are made from the rhizomes of *D. nipponica*, mainly composed of diosgenin. Through the clinical application for 302 cases of 18 clinical units in Sichuan Province, it was proved that Chuanlong guanxinning is effective for improving coronary blood supply, treating coronary heart disease, angina, palpitations, shortness of breath, choking, and high blood pressure [71].

In another clinical study, Chuanlong guanxinning was used for the treatment of 216 cases with coronary heart disease: 101 cases received one course and 115 cases received two treatment courses. The cases took 160 mg each time, three times a day over a course of 3 months. Among the 216 cases, 101 cases suffered angina before the treatment. Among them, 53 cases achieved effective results after the treatment, 93 cases’ symptoms were improved, and 14 cases had no effect. It was proved clinically that Chuanlong guanxinning had a significant effect in curing coronary heart disease and angina, with an effective rate of 91%. It was relatively effective for palpitations, shortness of breath and chest choking, with an effective rate of 77.3%. It can improve the insufficiency of coronary artery blood supply, hypertriglyceridemia and prevent the high blood pressure associated with coronary heart disease [120].

Wang [121] randomly selected 160 patients diagnosed with coronary heart disease and angina and evenly divided them into the treatment and control groups. The treatment group contained 42 males and 38 females with the average age of 59.3 years, among which 53 cases of unstable angina, 21 cases of stable angina, and 6 cases of old myocardial infarction were present. 80 cases with angina were cured with Weiaoxin with a total clinical effective rate of 90%, and the total effective rate of the electrocardiogram improvement was 65%. Moreover, adverse reactions such as bleeding, allergy and headache were not seen during the treatment.

### The treatment of chronic brucellosis

Brucellosis, especially chronic brucellosis is a common and frequently-occurring disease that has a long-term effect on public health and life. 302 cases with chronic brucellosis (186 male and 116 female) were treated with two dosage forms i.e., *D. nipponica* injection and syrup, by Gao [122]. The supervised No. 1 pill of traditional Chinese medicine was applied for the control group. The two preparations of *D. nipponica* can play the role in alleviating the clinical symptoms of chronic brucellosis and eliminating joint and nerve pain. The cure rate of the injection group was 32.14% with a basic cure rate of 38.4%; the cure rate of the syrup group was 24% with a basic cure rate of 2%; and the cure rate of the control group was 12.3% with a basic cure rate of 24.61%. The above data suggested that *D. nipponica* has clinical value for chronic brucellosis [122, 123].

Han [124] treated 231 cases of chronic brucellosis with an intramuscular injection of *D. nipponica*. The injection (4 mL) was applied once every other day or once a day, over a course of 10 days. The interval between the courses was 5–7 days, with 3 courses in total. The results showed an obvious curative effect on alleviating and eliminating arthralgia, headache, tiredness and pantalgia. Through the short-term and long-term clinical observations, the cure rate was 9.4%, the effective rate was 83.5% and the recurrence rate was 83.5%.

### The treatment of chronic bronchial asthma

Zhang et al. [92] used *D. nipponica* decoction and ligustrazine point injection simultaneously in 66 cases with bronchial asthma where the total effective rate was 92.42%, and the obvious effective rate was 56.06%. The control group contains 30 cases that were treated simultaneously with Ma xing Shi gan tang decoction and aminophylline point injection. The composition of the prescription includes *D. nipponica*, ephedra, Chinese thorowax root, *Fangfeng*, *Schisandra chinensis*, *Angelica sinensi*s, *Astragali* radix, *Fructus psoraleae*, *Rehmannia* root, *Scutellaria baicalensis*, *Rheum officinale,* and liquorice. The prescription could help to clear heat and eliminate phlegm, relieve asthma, nourish the kidney and sooth the liver, activate blood and relieve rheumatic pains. It was also verified by the modern pharmacology of traditional Chinese medicine that the above drugs could inhibit the adhesion and chemotaxis of the airway inflammatory cells, reduce inflammatory mediators and IgG content, and improve the inhibitive T-lymph cell functions.

By treating 37 cases of chronic bronchitis with the decoction of *D. nipponica* pills, the total effective rate was 86%, while for 142 cases it was 94%. The effective rates of the simple and resistance types were higher, i.e., 86% and 82%, respectively. The relief effect of cough, asthma and phlegm was favorable. The effect on the resistance type with repeated infection was very poor, and the effect of using *D. nipponica* alone for those with acute occurrence and respiratory tract infection caused by cold was unclear. After taking the *Scutellaria baicalensis* decoction simultaneously, the effect was significantly positive [4].

32 cases with serious bronchial asthma were treated by Lei [125] through the conventional treatment using western medicines. The clinical effect of serious bronchial asthma through Fei shu point injection of *D. nipponica* along with conventional treatment of Western medicines was observed for 2 weeks. The results showed that the total effective rate was 78.13% and there were different degrees of improvement for the lung function indices of patients. Thus, the point injection of *D. nipponica* was relatively effective and safe for the treatment of serious bronchial asthma.

### The treatment of lipoma

*Dioscorea nipponica* (250 g) was soaked at 60 °C in wine (2000 mL) for half a month and administered to a patient with scapulohumeral periarthritis. Though the result was not clear for scapulohumeral periarthritis, the two-decade old egg-sized lipoma on the hip of the patient had become soft and small unexpectedly. After oral administration for 3 months, the lipoma had completely disappeared. Therefore, the combination of *D. nipponica* and wine was considered to be optimal as the former has the effect of activating blood and the latter has the function of dredging collaterals [126].

## Toxic and side effects

Adverse reactions of *D. nipponica* include diarrhoea, astriction, abdominal discomfort accompanied by nausea, emesis, stomatitis, epistaxis, dizziness, increased menstrual volume, swelling at the injection site, blurred vision and temporary increase of glutamate pyruvate transaminase (GPT). These symptoms would disappear once the drug use is suspended and during the treatment, all these symptoms should be noted [5].

The toxicity of the water-soluble TSRDN varies through administration routes. In mice, the maximum tolerated dose (MTD) through oral route was 15.6 g/kg and the median lethal dose (LD_50_) was 2.21 ± 0.14 g/kg. The LD_50_ of TSRDN intravenous injection was 750 mg/kg while that of its pure product was 406–425 mg/kg. 60–180 mg/kg daily gavage for mice was conducted for 7 weeks consecutively, and the result showed no clear influence on the hemogram, liver and spleen function. Through visual observation of the heart, liver, spleen, lung, kidney, adrenal gland and other key organs, no pathologic change had been found. According to Wang Miaofang et al. [127], the main constituent of the Wei Ao Xin medicine was water-soluble total saponin which could cause an increase in the release of bradykinin and prostaglandins E2 that leads to dry cough, due to the stimulation of the respiratory tract.

Liang et al. [128] used the gavage and hypodermic injection to observe the acute toxic effect of TSRDN in mice. The MTD of the TSRDN gavage for 7 days consecutively after one-time drug administration was 0.42 g/kg/d. The LD_50_ of TSRDN caused by the gavage was not measured by the result but equivalent to 1355 times the MTD of the daily clinical dose of adults. The LD_50_ of hypodermic TSRDN injection of mice was 0.17 g/kg, indicating that the oral drug administration of TSRDN was relatively safe. In addition, TSRDN may have a certain influence on the gastrointestinal tract of mice, which needs further long-term toxicity observations.

Zhou et al. [129] used the in vitro 3-(4,5-dimethythiazol-2-yl)-2,5-diphenyl tetrazolium bromide (MTT) method to detect the alanine aminotransferase (ALT), alkaline phosphatase (ALP) and lactate dehydrogenase (LDH) content in the liver due to dioscin toxicity. According to experimental research on mice, after 7 days of dioscin administration (10 mg/kg), the aspartate aminotransferase (AST) and LDH levels in Kunming mouse serum have increased. The liver of one mouse showed wide edema of hepatocytes, and two mice showed occasional meganucleus hepatocyte, implying that large doses of dioscin might be harmful to the liver to some extent. So, the clinical use of drugs with dioscin should be cautious, especially for patients with poor liver function.

## Discussion

The significance of *D. nipponica* in Traditional Chinese Medicine has led to the isolation of numerous steroid saponins during the last few years possessing furostane-, spirostane-, and pregnane-type aglycones. The isolation was done following standard procedures, while the structure elucidation was performed with the help of advanced spectroscopic (NMR, MS, etc.) techniques. Numerous in vitro and in vivo assays helped researchers to characterize several pharmacologically active steroid saponins in *D. nipponica* possessing anti-tumor, immunoregulatory, analgesic, anti-microbial, anti-platelet, anti-ischemic, anti-anginal, anti-asthmatic, anti-inflammatory, and anti-allergic activities. These comprehensive pharmacological investigations have revealed that the therapeutic applications of *D. nipponica* are substantial and invaluable.

However, the clinical trials are still very few and more evidence-based clinical trials are required to study the efficacy of *D. nipponica*. Its toxic effects on liver should be taken into consideration before calculating the dose and preparing the dosage regimens in clinical therapeutic applications. Hence, the mechanisms involved in the toxic and side effects, as well as the safety profile of the *D. nipponica* extracts, need to be studied well and the toxic metabolites need to be characterized. In addition, synergism and reduced toxic effects has been achieved through the combination of *D. nipponica* with other herbal medicines. Further, the major drawbacks of potent chemotherapeutic drugs are toxicity to normal tissues and drug resistance. In this context, dietary supplementation, as well as phytotherapeutic agents with high anti-cancer activity and less toxicity, has been considered as potential candidates for their capacity to enhance the efficacy of anti-cancer drugs.

*Dioscorea nipponica* has high medicinal value, extensive utility and encouraging market prospects. Various synthetic contraceptives and most steroid hormone drugs in the world are made from dioscin. Annually, there is a worldwide requirement for 8000 tons of dioscin, but actually, only 3000 tons can be produced [130]. With unique medicinal values, *D. nipponica* has become a rare resource snapped up by biopharmaceutical enterprises. As wild resources are nearly exhausted, steroid medicinal resource plants are in short supply, while the artificial cultivation of *D. opposita* is in face of degenerated quality and low output, making the raw material of *D. nipponica* extremely insufficient [131, 132].

### Measures proposed to deal with the exhaustion of *D. nipponica* resources

#### Strengthening the protection of wild *D. nipponica*

The development and utilization of *D. nipponica* resources are relatively slow, but its application scope and demand have been increasing with people’s deepening understanding. The gradual reduction, as well as free utilization of wild resources, has resulted in the failure of meeting the market demand. In addition, *D. nipponica* grows mainly on the hillsides, which allows it to be exploited easily by humans.

#### Strengthening the study on *D. nipponica* cultivation techniques

The industrial cultivation of *D. nipponica* has a short history; so, many problems need to be resolved urgently. In recent years, numerous studies on the cultivation techniques of *D. nipponica* have been conducted. The original dimming production skills have been changed, which has led to the significant reduction of production costs and dramatic increase in output. According to the biological characteristics of *D. nipponica*, the age of harvesting and production should be prolonged appropriately to get high outputs. Since the percentage of active constituents varies with different cultivation techniques and geography; the cultivation should be standardized to yield chemically standard herb. Secondly, there is a need for the development of the plant strains which are resistant to various plant diseases.

#### Establishing normalized and intensified planting bases of *D. nipponica*

The steroid content of *D. nipponica* is taken as the index, which verified that cultivated *D. nipponica* has relatively higher quality than wild ones. The main reason is that the synthesis of steroid saponins is closely related to the illumination. As a herbaceous twiner, *D. nipponica* needs to climb on other plants in natural habitat, so it has weak illumination. The cultivated *D. nipponica* receives full illumination which is beneficial to the synthesis of dioscin, thereby providing a favorable technical support for the culture prospects. In cultivated plants, the content of diosgenin can be much higher than wild products. Through various breeding and culture techniques, the content of diosgenin could be improved continuously. As a cash crop for farming industry adjustment in rural areas, the development of *D. nipponica* has huge potential. New culture techniques of *D. nipponica*, and comprehensive technical developments like saponin extraction and in-depth processing, have provided a more extensive development prospect, which is conductive to the economic and social benefits.

### Problems associated with the extraction of bioactive compounds

The following are some of the problems faced during the extraction of bioactive compounds from *D. nipponica*: The rate of extraction and separation are generally low, and only the extraction of diosgenin is given priority, while other bioactive compounds such as the starch and tannin are neglected, which results in the waste of valuable resources [132]. The organic solvent extraction method is generally used, but the organic solvents are toxic and pollutants to the environment. Hence, the following aspects should be noted in future research and development: The extraction technique can be optimized along with rational utilization of the bioactive compounds; the cost of critical fluid extraction can be reduced by improving the extraction techniques. Thus, these measures could effectively resolve the main problems like low extraction rate and environmental pollution.

## Conclusion and future perspectives

This review has summarized the botanical, phytochemical and pharmacological properties, as well as the clinical applications of the traditional Chinese medicinal plant *D. nipponica.* The steroid saponins occurring in the plant have the effect of improving cardiovascular system, regulating immune function, reducing swelling, relieving phlegm, cough, and asthma, as well as preventing inflammation and pain. However, there is still no clear classification and in-depth studies on the chemical components of *D. nipponica* and their activities, so the pharmacological mechanism has not been understood well. Research on the pharmacological activities is still in the animal experiment phase with a few reports on the clinical applications; therefore, further studies are needed [73].

Due to the shortage of wild *D. nipponica* resources, the biological features, cultivation, development and utilization should be strengthened. By increasing the production and decreasing production costs, the development of the agriculture should be intensified. The comprehensive utilization of all the plant products will help to promote the cultivation as well as the protection of wild resources. With high economic value, *D. nipponica* is an abundant resource of bioactive compounds in China. As people are attaching greater importance to the human health, the research on *D. nipponica*, mainly its water-soluble active ingredients should be strengthened. Further research is necessary to determine the chemical structure of the compounds, which may be specifically applied to pharmacology and clinical applications. If effective drugs can be made using advanced synthetic techniques, they will play a key role in enhancing health. Therefore, this review would be valuable for promoting the development of *D. nipponica* as a safe modern pharmaceutical as well as for improving its clinical uses.

## Abbreviations

2,4-DNFB: dinitrofluorobenzene
6-Keto-PGF1α: 6-ketone-prostaglandin F1α
ALP: alkaline phosphatase
ALT: alanine aminotransferase
ANAE: nonspecific acid esterase
AST: aspartate aminotransferase
BALF: bronchoalveolar lavage fluid
CIA: collagen-induced arthritis
CK: creatine kinase
ConA: concanavalin A
ELISA: enzyme-linked immunosorbent assay
EOS: eosinophilic granulocyte
FCM: flow cytometry
FT-207: 1-(2-tetrahydrofuryl)-5-fluorouracil
Glc: *β*-d-glucopyranosyl
GPT: glutamate pyruvate transaminase
HPLC: high performance liquid chromatography
I/R: ischemia-reperfusion
LDH: lactate dehydrogenase
LDH: lactate dehydrogenase
LPS: lipopolysaccharide
MDA: malondialdehyde
MTD: maximum tolerated dose
MTT: 3-(4,5-dimethythiazol-2-yl)-2,5-diphenyl tetrazolium bromide
MVD: microvessel density
NIS: sodium-iodide symporter
NO: nitric oxide
OVA: ovalbumin
P.B: prosapogenin B
PAF: platelet-activating factor
RA: rheumatoid arthritis
RDN: *Rhizoma Dioscorea nipponica*
Rha: *α*-l-rhamnopyranosyl
RP-HPLC: reversed-phase HPLC
SOD: superoxide dismutase
SPF: specific-pathogen-free
SRBC: sheep red blood cell
TLC: thin layer chromatography
TSRDN: total saponins of *Rhizoma Dioscreae nipponica*
TXB2: thromboxane B2
VEGF: vascular endothelial growth factors
WERDN: rhizome water extracts of *D. nipponica*
α-SMA: α-smooth muscle actin
